# Role of SdiA on Biofilm Formation by Atypical Enteropathogenic *Escherichia coli*

**DOI:** 10.3390/genes9050253

**Published:** 2018-05-15

**Authors:** Hebert F. Culler, Samuel C. F. Couto, Juliana S. Higa, Renato M. Ruiz, Min J. Yang, Vanessa Bueris, Marcia R. Franzolin, Marcelo P. Sircili

**Affiliations:** 1 Laboratory of Genetics, Butantan Institute, São Paulo 05503-900, Brazil; hculler2@hotmail.com (H.F.C.); samuel.couto@butantan.gov.br (S.C.F.C.); juliana.higa@butantan.gov.br (J.S.H.); remelloruiz@gmail.com (R.M.R.); min.yang@butantan.gov.br (M.J.Y.); vanessa.bueris@butantan.gov.br (V.B.); 2 Laboratory of Bacteriology, Butantan Institute, São Paulo 05503-900, Brazil; marcia.franzolin@butantan.gov.br

**Keywords:** atypical enteropathogenic *Escherichia coli*, biofilm formation, *sdiA* gene, acyl homoserine lactone, confocal scanning laser microscopy

## Abstract

Atypical enteropathogenic *Escherichia coli* are capable to form biofilm on biotic and abiotic surfaces, regardless of the adherence pattern displayed. Several *E. coli* mechanisms are regulated by Quorum sensing (QS), including virulence factors and biofilm formation. Quorum sensing is a signaling system that confers bacteria with the ability to respond to chemical molecules known as autoinducers. Suppressor of division inhibitor (SdiA) is a QS receptor present in atypical enteropathogenic *E.* *coli* (aEPEC) that detects acyl homoserine lactone (AHL) type autoinducers. However, these bacteria do not encode an AHL synthase, but they are capable of sensing AHL molecules produced by other species, establishing an inter-species bacterial communication. In this study, we performed experiments to evaluate pellicle, ring-like structure and biofilm formation on wild type, *sdiA* mutants and complemented strains. We also evaluated the transcription of genes involved in different stages of biofilm formation, such as *bcsA*, *csgA*, *csgD*, *fliC* and *fimA.* The *sdiA* mutants were capable of forming thicker biofilm structures and showed increased motility when compared to wild type and complemented strains. Moreover, they also showed denser pellicles and ring-like structures. Quantitative real-time PCR (qRT-PCR) analysis demonstrated increased *csgA, csgD* and *fliC* transcription on mutant strains. Biofilm formation, as well as *csgD*, *csgA* and *fimA* transcription decreased on wild type strains by the addition of AHL. These results indicate that SdiA participates on the regulation of these phenotypes in aEPEC and that AHL addition enhances the repressor effect of this receptor on the transcription of biofilm and motility related genes.

## 1. Introduction

Enteropathogenic *Escherichia coli* (EPEC) is a major cause of diarrhea in children in developing countries [1,2]. EPEC produces a characteristic histopathologic lesion known as attaching and effacing (A/E) on the intestinal mucosa [3,4,5,6]. The A/E lesion results from intimate bacterial adherence to the enterocytes, followed by local microvillus effacement and accumulation of polymerized actin of the cytoskeleton underneath adherent bacteria, forming pedestal-like structures [7]. EPEC can be divided in two groups: typical EPEC (tEPEC) and atypical EPEC (aEPEC) [3]. tEPEC strains contain the EAF (EPEC adherence factor) plasmid, which is absent in aEPEC [5]. Recent epidemiological studies indicate that aEPEC is more prevalent than tEPEC in both developed and developing countries [8]. The aEPEC strains are genetically related to the enterohemorrhagic *E. coli* (EHEC) strains and both are considered emerging pathogens [5].

Diarrhea caused by aEPEC is usually more persistent than diarrhea caused by other pathogens and these bacteria seem to remain in the intestine for longer periods than other diarrheagenic *E*. *coli* (DEC) [8]. This persistence can be related to the ability to form biofilm, both on the environment and in the host. Moreira et al. [9] showed that tEPEC strains are capable of forming biofilm in abiotic surfaces, glass and polystyrene and in biotic surfaces such as pre-fixed cells. More recently, Weiss-Muszkat et al. [10] demonstrated that O55:H7 aEPEC strain form biofilm at 26 °C and Culler et al. [11] showed that aEPEC from different serotypes form biofilm at 37 °C in abiotic surfaces and pre-fixed cells. In addition, Hernandes et al. [12] reported that type one fimbriae are involved in biofilm formation by this pathotype.

In order to biofilm formation to occur, bacteria demand certain structures that aid on its anchorage to the substratum and several studies have described the importance of curli fimbriae [13,14], type one fimbriae [12], motility [15,16] and cellulose [17,18] in the biofilm formation by *E. coli* strains.

Saldaña et al. studied the contribution of curli fimbriae and cellulose on the adherence of prototype tEPEC (E2348/69) and EHEC (EDL933) strains by the use of mutants lacking either cellulose (*bcsA* gene) and curli fimbriae (*csgA* gene), as well as a double mutant (*bcsA*/*csgA*). The results showed that both structures are important for proper adhesion by the prototype strains, indicating a synergism between cellulose and curli fimbriae. Moreover, the authors also verified that the overexpression of curli and cellulose enhance biofilm formation by EHEC.

In work conducted by Weiss-Muszkat et al. [10], it was reported that *csgFG* mutants are incapable of forming pellicle and ring-like structure, indicating that curly may be fundamental for the formation of these structures. Hernandes et al. [12] also demonstrated the importance of *fimA* on the formation of ring-like structures in glass tubes after 48 h at 37 °C. These results suggest that the formation of this structure is probably under control of a multifactorial process, involving the expression of several structures. On the other hand, Nascimento et al. [19] verified that an aEPEC O119:HNT clinical strain was able to form pellicle at 26 °C even in the absence of curli expression, indicating that other factors might be involved in its formation on this condition.

Quorum sensing (QS) is a density dependent regulatory mechanism mediated by the accumulation of signaling molecules produced by bacteria and is related to transcriptional regulation of several genes, including those involved in biofilm formation, bacterial adhesion, host colonization and virulence factors [9,20,21].

*Escherichia*, *Salmonella*, *Klebsiella*, *Shigella* and *Enterobacter* encode a transcriptional activator protein-LuxR homologue Quorum sensing receptor—called suppressor of division inhibitor (SdiA)—which is an acyl homoserine lactone (AHL) receptor. Even though these bacteria do not encode an AHL synthase [22,23,24,25,26,27,28], SdiA can detect AHL molecules produced by other bacterial genus [27]. Similar to other LuxR type proteins, SdiA contains a helix-turn-helix (HTH) DNA binding site in its C-terminal region and an AHL binding domain in its N-terminal region [24,29,30].

SdiA presents specific characteristics that differentiates it from other LuxR type receptors. In a recent study, Kim et al. [30] verified that SdiA binds to the promoter region of cell division operon *ftsQP2* even in the absence of AHL, suggesting a functional role for SdiA independent of molecule signaling. Similar results were reported by other groups [31,32,33,34,35,36].

Shimada et al. [34], demonstrated that *sdiA* transcription is regulated by several transcription factors, among them ArcA, CpxR, OmpR, RcsB and TorR are two-component systems that repress *sdiA* transcription.

In EHEC, *Enterobacter* and *E. coli* K-12 BW25113, *sdiA* participates in the regulation of a number of virulence factors such as curli production, biofilm formation, adhesion on epithelial cells and motility [26,27,35]. However, in aEPEC the role of SdiA is not known.

In this work, we demonstrated the involvement of *sdiA* on biofilm, pellicle and ring-like formation, cellulose production, curli and type one fimbriae expression. We also analyzed the influence of *N*-(3-oxohexanoyl)-DL-homoserine lactone (3O-C6-DL-HSL) and *N*-octanoyl-DL-homoserine lactone (C8-DL-HSL) on adhesion and biofilm formation.

## 2. Materials and Methods

### 2.1. Bacterial Strains, Culture Conditions and Plasmids

Strains and plasmids used in this work are listed in Table 1. ONT:H25 aEPEC strain was isolated from patients with diarrhea and were genetically and phenotypically characterized in previous studies as (intimin [*eae*+]/EPEC adherence factor [EAF]-/Shiga toxin [*stx*]-/bundle forming pilus [*bfpA*-]) [37], aggregative and localized adherence-like pattern in HEp-2 cells [38] and highly biofilm formation [11]. Atypical EPEC strain was grown in Luria-Bertani (LB) broth at 37 °C for 18 h, unless otherwise stated. Culture media was supplemented with ampicillin (100 µg/mL), kanamycin (50 µg/mL) and/or chloramphenicol (20 µg/mL) when needed. Two µM of *N*-(3-oxohexanoyl)-DL-homoserine lactone (3O-C6-DL-HSL) and *N*-octanoyl-DL-homoserine lactone (C8-DL-HSL) (Sigma-Aldrich Co., St. Louis, MO, USA) dissolved in dimethyl sulfoxide (DMSO) were used when needed. Since AHLs were dissolved in DMSO, we used this molecule also as a negative control in the experiments when needed.

### 2.2. Recombinant DNA Techniques

PCR reactions, plasmid purification, digestion with restriction enzyme, ligation, bacterial transformation and agarose gel electrophoresis were performed according to Sambrook and Russell [39]. The *sdiA* mutant strain (HFC01) was obtained by homologous recombination replacing *sdiA* gene of aEPEC wild type ONT:H25 strain by a cloranfenicol cassette through λ Red system [40]. All oligonucleotides primers (Life Technologies/Invitrogen, Carlsbad, CA, USA) used in this work are listed in Table 2. Mutant strain was complemented with pBAD/Myc-His A containing *sdiA* gene sequence inserted into the *Xho*I and *EcoR*I cloning site. Mutation and complementation were confirmed by sequencing.

### 2.3. Congo Red Binding and Cellulose Assay

Bacterial strains were grown in 2% agar plates (1% casamino acid; 0.15% yeast extract; 0.005% magnesium sulfate), supplemented with 40 µg/mL of Congo red (CR) and 20 µg/mL of Coomassie brilliant blue G250 (Merck KGaG, Darmstadt, Germany) at 26 °C and 37 °C for 24 and 48 h, to identify curli expression. Cellulose assay was performed with bacterial strains grown in Luria Bertani no Salt (LBNS) agar supplemented with 100 µg/mL of Calcofluor (Sigma-Aldrich Co.) for 24 and 48 h at 37 °C and 26 °C. Cellulose production was detected by UV light at 366 nm.

### 2.4. Pellicle and Ring-Like Structure Formation

To study pellicle and ring-like structure formations, 30 µL from an overnight culture grown in LB at 37 °C were subcultured in 3 mL LB for 72 h, either under static or shaking conditions (150 rpm) at 26 °C or 37 °C. A pellicle and ring-like formation in an air-liquid-glass interface was analyzed and photographed with a digital camera.

### 2.5. Biofilm Assay with Crystal Violet

Biofilm formation on polystyrene surface by aEPEC strains was assayed following the method described by Sheikh et al. [43] with slight modifications. Overnight bacterial cultures were grown in LB under static conditions and were inoculated into fresh LB at a 1:100 dilution in 96-well plates in a final volume of 200 µL. The plates were incubated at 26 °C and 37 °C for 24 h and 72 h. After the incubation period, the culture medium was aspirated, and the preparation was washed with 1× PBS. Biofilm was fixed with 200 µL of 75% ethanol per well for 10 min, washed three times to remove the ethanol and stained with 0.5% CV for 5 min. After PBS washings, the plates were air-dried, and the Crystal Violet (CV) was solubilized by the addition of 200 µL of 95% ethanol per well. After 2 min at room temperature, 150 µL of the solution was transferred to a microtiter plate and the absorbance was determined with an enzyme-linked immunosorbent assay (ELISA) plate reader (Multiskan EX, Thermo Fisher Scientific, Waltham, MA, USA) at 595 nm. All assays were performed in triplicate. For the AHL assays, DMSO, 3O-C6-DL-HSL or C8-DL-HSL were added on LB prior to bacteria inoculation.

### 2.6. Confocal Scanning Laser Microscopy

Microscopic analysis of biofilm formation on glass surface was performed according to the methodology described by Culler et al. [11]. Overnight bacterial cultures were grown in LB under static conditions and inoculated into fresh LB medium in a 1:100 dilution in 24-well cell culture plates with glass coverslips, in a final volume of 1 mL. After 24 h of incubation at 37 °C, the culture medium was aspirated, the preparation was washed with 1× PBS and fixed with 4% formalin in PBS, for 18 h at 4 °C. The plates were washed again with PBS containing 0.2% bovine serum albumin (PBS-BSA), permeabilized with PBS with 0.1% Triton X-100 for 5 min and washed with 0.2% PBS-BSA. Preparations were incubated with propidium iodide (PI, Molecular Probes, Eugene, OR, USA) for 45 min. After four times washing for 5 min with 0.2% PBS-BSA, preparations were examined under confocal scanning laser microscopy (CLSM) (LSM 510 meta, Carl Zeiss, Oberkochen, Germany), using a 570–719 filter and wavelength of 543 nm and 630× magnification.

### 2.7. Fluorescent Actin Staining

Fluorescent actin staining (FAS) assay on HeLa cells was performed according to the methodology described by Knutton et al. [6]. Previously prepared plates containing 5 × 10^4^ HeLa cells were inoculated with 4:100 dilutions of bacteria in Dulbecco’s Modified Eagle’s Medium (DMEM) medium. After 6 h of incubation at 37 °C, the culture medium was aspirated, and the next steps were carried out according to the methodology described above, with the addition of the following steps. The preparations were incubated with Alexa Fluor 488-Phalloidin (Molecular Probes) and PI for 45 min. After four-time washing during 5 min with 0.2% PBS-BSA, preparations were examined under LSM 510 Meta (Carl Zeiss, Oberkochen, Germany) confocal microscope.

### 2.8. Confocal Scanning Laser Microscopy of Ring-Like Structure in Air-Liquid-Glass Interface

Qualitative analysis of ring-like structure formation in air-liquid-glass interface was performed through confocal microscopy. A 24-well cell culture plate was filled with 300–500 µL of 3% sterile agarose to vertically hold the glass coverslips. One mL of LB broth was added to each well and a 1:100 dilution of bacterial culture was inoculated. After 48 h at 37 °C the coverslips were stained with PI, as described above.

### 2.9. Motility Assays

Motility assays were performed as described by Sperandio et al. [44]. Bacterial strains were previously grown in LB for 18 h at 37 °C. With the use of a bacteriological needle, the strains were inoculated on motility agar plates (0.3% agar, 1% tryptone and 0.25% NaCl). The motility halo was measured after 8 h of incubation at 37 °C.

### 2.10. RNA Extraction and Quantitative Real-Time PCR

Total RNA from ONT:H25 wild type, *sdiA* mutants and complemented strains was extracted using RNAprotect^®^ Bacteria Reagent and RNeasy^®^ Mini Kit (QIAGEN, USA) according to the manufacturer’s instructions. Strains were grown at 37 °C until OD 0.6 in LB broth. DMSO and 3O-C6-DL-HSL were added when needed. RNA transcription was quantified using the Applied Biosystems (Foster city, CA, USA) ABI 7500 Fast real time PCR system. One microgram of RNA was treated with DNase (Promega Corporation, Madison, WI, USA) and the total RNA was converted into complementary DNA (cDNA) by reverse transcription (Applied Biosystems). Next, the cDNA was added to a mixture containing SYBR Green PCR Master Mix (Applied Biosystems) and previously validated primers (Integrated DNA Technologies-IDT, Coralville, IA, USA). All primers used in qRT-PCR reactions are listed in Table 2 and were validated for amplification efficiency and template specificity. The data was normalized to a RNA polymerase alfa (*rpoA*) endogenous control and analyzed using the comparative critical threshold method [41]. The assays were carried out in triplicate and data was collected by 7500 software v2.0.5 (Applied Biosystems). The expression levels of the target genes were presented as fold changes over the expression level of wild type aEPEC. Error bars represent the standard deviation of the ∆∆*Ct* value.

### 2.11. Statistical Analysis

Statistics were performed using Student’s unpaired *t*-test. Differences were considered significant when *p*-value < 0.05.

## 3. Results

### 3.1. Deletion of sdiA Alters Curli Expression

**Figure 1:**
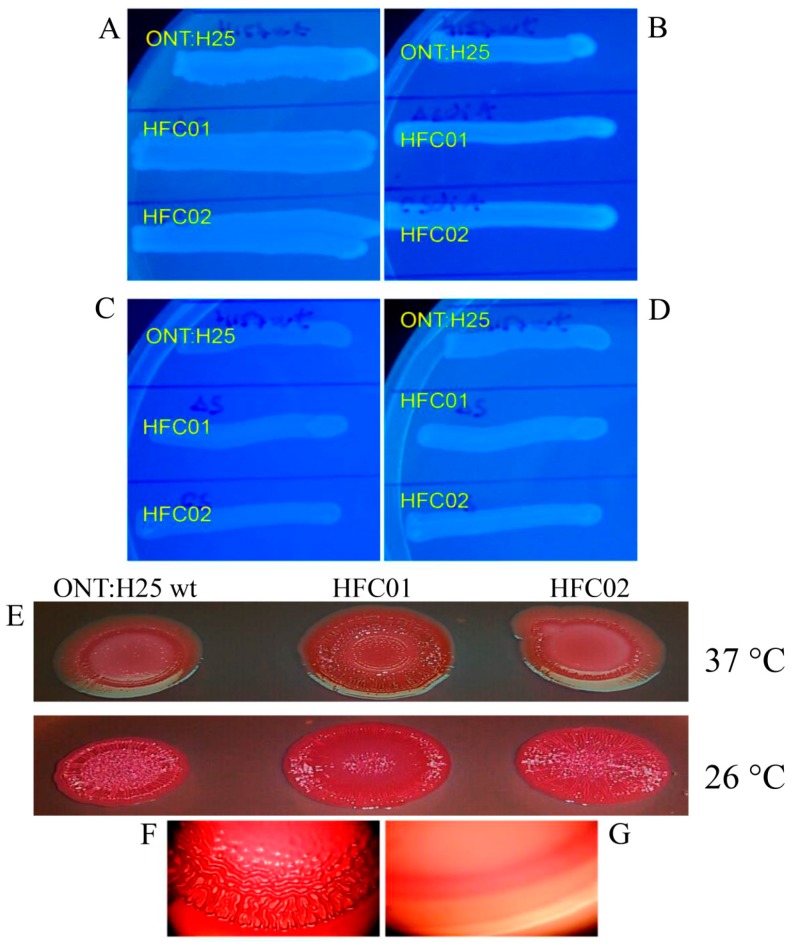
Cellulose production by ONT:H25, *sdiA* mutant strain (HFC01) and ONT:H25 wild type and complemented strain (HFC02), strains on LBNS agar plates supplemented with calcofluor. (**A**,**C**) 24 h of incubation at 37 °C and 26 °C, respectively; (**B**,**D**) 48 h of incubation at 37 °C and 26 °C, respectively. Curli production by ONT:H25, HFC01 and HFC02 strains on LBNS agar plates supplemented with Congo Red and Coomassie brilliant blue, after 24 h at 26 °C. (**E**) ONT:H25, HFC01 and HFC02; (**F**) rdar colonie morphotype. (**G**) smooth colonie morphotype.

Coloring differences of strains grown in CR and Coomassie Brilliant Blue plates are due to levels of fimbriae curli expression on the bacterial cell surface. Therefore, the bacterial strains were grown in CR and Comassie Blue plates, which indicate cellulose production besides fimbriae curli expression. The ONT:H25 wild type strain and complemented strain (HFC02) displayed red colored colonies and the *rdar* (red, dry and rough) morphotype after 24 h of growth at 26 °C. HFC01 showed roughness mainly in the center of the colony (Figure 1E). The HFC01 strain grown at 37 °C presented intense red colony formation and the *rdar* morphotype in contrast to the wild type and complemented strain after 24 h of incubation.

In this study, the analysis through Calcofluor plates showed no visual differences in the production of cellulose by the wild type, mutant and complemented strains at 24 or 48 h of incubation (Figure 1). However, all the strains grown at 26 °C (Figure 1C) showed decreased cellulose production in comparison to strains grown at 37 °C (Figure 1A,C) after 24 h of incubation.

### 3.2. sdiA Suppresses Biofilm Formation

In order to verify the influence of *sdiA* gene deletion on biofilm formation by aEPEC, ONT:H25 *sdiA* mutants were evaluated through colorimetric assay with CV.

**Figure 2:**
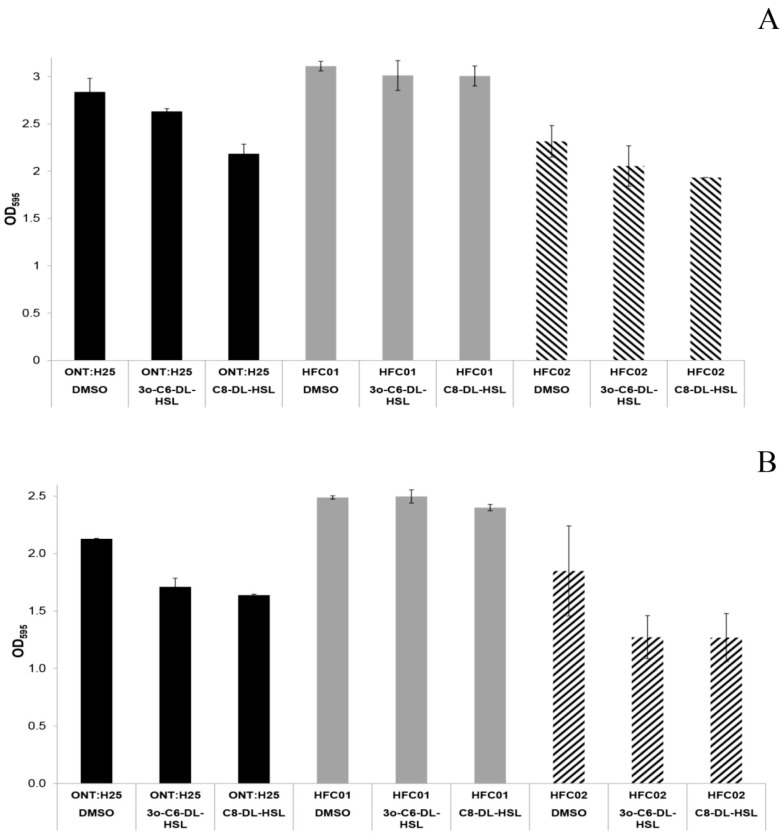
(**A**) Biofilm formation by ONT:H25, HFC01 and HFC02 after 24 h of incubation at 26 °C in Luria-Bertani (LB) with dimethyl sulfoxide (DMSO), 3O-C6-DL-HSL or C8-DL-HSL. Statistic bars indicate standard deviation. (**B**) Biofilm formation by ONT:H25, HFC01 and HFC02 after 24 h of incubation at 37 °C in LB with DMSO, 3O-C6-DL-HSL or C8-DL-HSL. Statistic bars indicate standard deviation.

Mutant strains were able to form thicker biofilm in comparison to wild type and complemented strains. A Significant statistical difference was observed in strain HFC01 (*p*-value < 0.05) (Figure 2). 

Considering that the SdiA receptor is capable of recognizing AHL molecules and regulate gene transcription of several bacterial species, we evaluated the influence of these molecules on adhesion and biofilm formation by aEPEC ONT:H25 wild type, *sdiA* mutant and complemented strains.

The addition of 3O-C6-DL-HSL and C8-DL-HSL in ONT:H25 strains significantly decreased (*p*-value < 0.05) biofilm formation by wild type and complemented strains. This was not observed in *sdiA* mutant strains, indicating that SdiA responds to AHLs and negatively regulates biofilm formation in these aEPEC strains.

### 3.3. Deletion of sdiA Gene Increases Pellicle Formation in Air-Liquid Interface and Ring-Like Structure in Air-Liquid-Glass Interface

**Figure 3:**
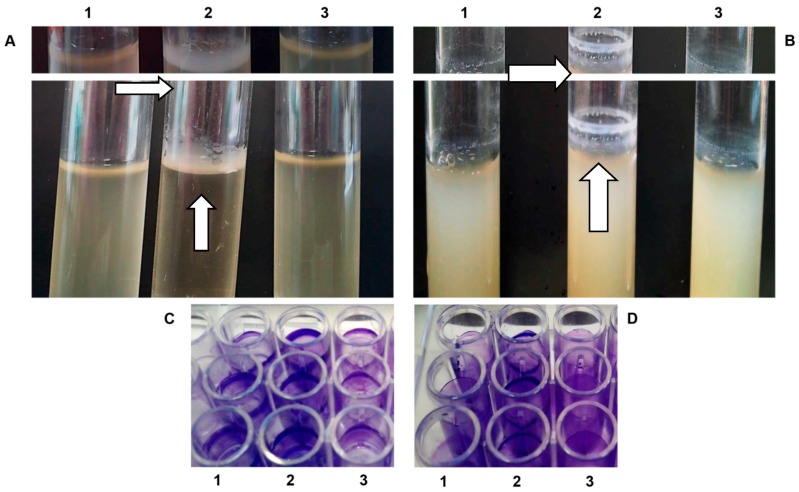
Pellicle and ring-like structure formation by ONT:H25, HFC01 and HFC02. (**A**) Pellicle formation on air-liquid interface in 24 h at 37 °C in LB, arrows indicate pellicle and ring-like structure formation; (**B**) Ring-like structure formation on air-liquid-glass interface in 72 h at 37 °C in LB, arrows indicate pellicle and ring-like structure formation; (**C**) Ring-like structure formation in 96-well plate after 72 h at 26 °C. **1**, ONT:H25; **2**, HFC01; **3**, HFC02. (**D**) Ring-like structure formation in 96-well plate after 72 h at 37 °C in LB. **1**, ONT:H25; **2**, HFC01; **3**, HFC02.

The HFC01 strain formed a thick pellicle in the air-liquid interface after 24 h of incubation at 37 °C (Figure 3A) and at 26 °C [45]. Wild type and complemented strains were considered negative for this phenotype (Figure 3A). Regarding ring-like structure formation, the HFC01 strain displayed a thick layer in the tube wall after 72 h of incubation at 37 °C. This thick layer was not observed in the wild type and complemented strains (Figure 3B,D). On the other hand, at 26 °C all the strains formed a similar ring-like structure, with the mutant strain forming a slightly thicker structure (Figure 3C).

### 3.4. Presence of sdiA and AHL Result in Different Biofilm Architectures

**Figure 4:**
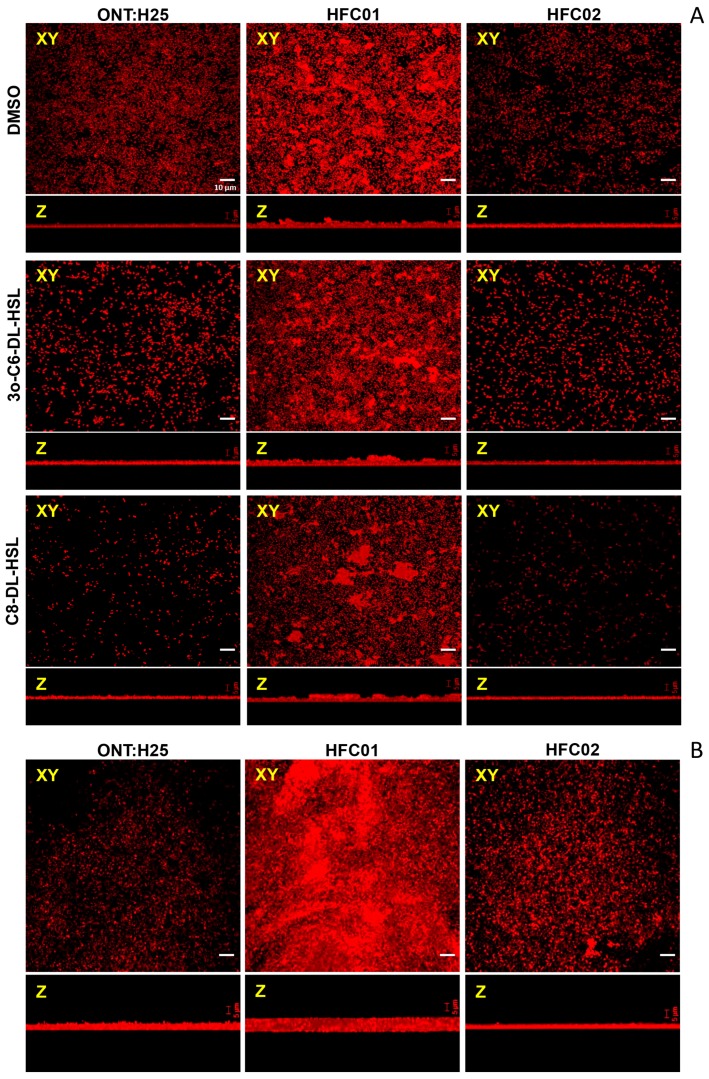
(**A**) Biofilm formation by ONT:H25, HFC01 and HFC02 after 24 h of incubation at 37 °C on LB supplemented with DMSO, 3O-C6-DL-HSL and C8-DL-HSL. (**B**) Biofilm formation on air-liquid-glass interface by ONT:H25, HFC01 and HFC02 after 48 h of incubation. **XY**—*xy* axis, **Z**—*z* axis from XY. Bacteria were stained with Propidium Iodide (red). 630× magnification.

In order to quantitatively evaluate the effect of *sdiA* deletion on biofilm formation, CLSM assay was performed. CLSM revealed a characteristic mature biofilm architecture on abiotic surface, where it was possible to observe pillars in the ONT:H25 *sdiA* mutant strain in every treatment applied (DMSO, C8-DL-HSL and 3O-C6-DL-HSL) (Figure 4A). Wild type and complemented strains displayed a thin biofilm formation and upon AHL addition the bacterial monolayer was diminished. These results corroborate with those observed in CV assay, indicating that SdiA responds to AHL potentiating its suppressor effect on biofilm formation.

We also analyzed the ring-like structure formation in air-liquid-glass interface of ONT:H25, HFC01 and HFC02 through CLSM [11]. As seen in Figure 4B, HFC01 strain formed a thicker biofilm structure, in which even the characteristic pillars could be observed.

### 3.5. AHL Addition Decreases aEPEC Adhesion

**Figure 5:**
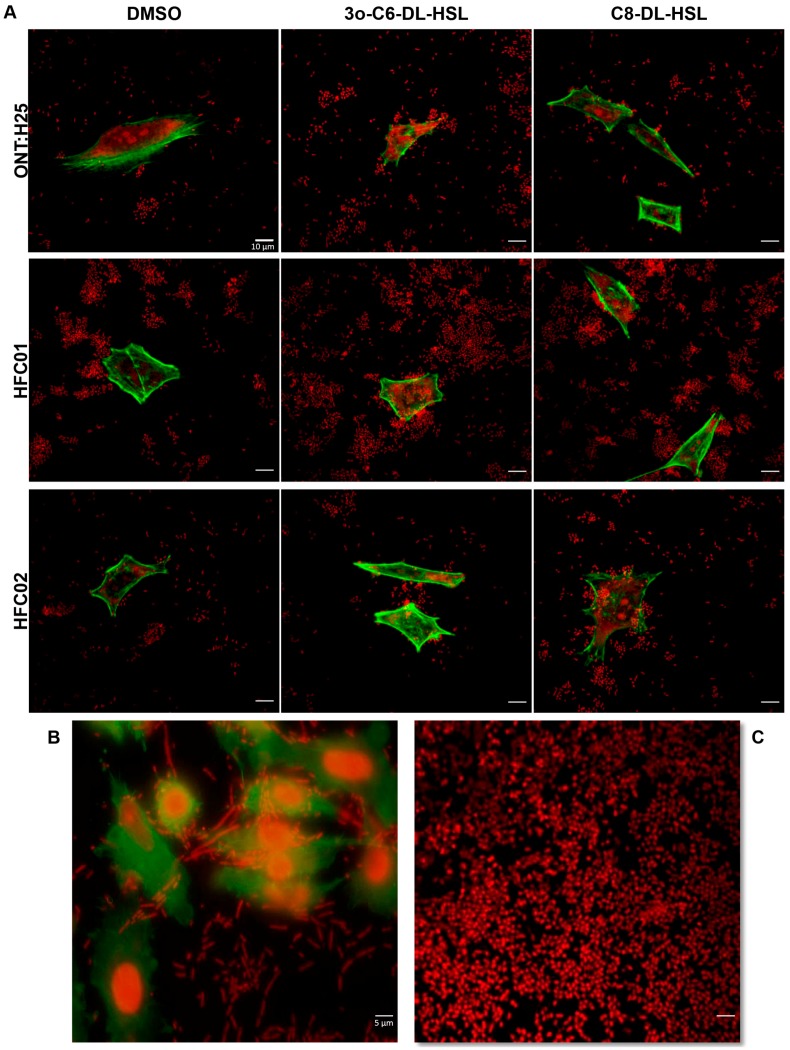
(**A**) Fluorescent actin staining assay of ONT:H25, HFC01 and HFC02 in HeLa cells in Dulbecco’s Modified Eagle Medium (DMEM) supplemented with DMSO, 3O-C6-DL-HSL and C8-DL-HSL after 6 h of incubation at 37 °C. (**B**) Long filament-like morphology displayed by bacteria in the presence of HeLa cells. (**C**) Bacteria in the absence of HeLa cells. HeLa cells were stained with Alexa Fluor–Phalloidin (green) and nucleic acids were stained with Propidium Iodide (red). (**A**), 630× magnification; (**B**,**C**), 1000× magnification.

The decrease of biofilm formation due to AHL addition led us to investigate the influence of these molecules in aEPEC adhesion in HeLa cells. Assays were performed with DMEM supplemented with 1% mannose, which has affinity to type 1 fimbriae and blocks its binding to cellular or abiotic surfaces. ONT:H25 adhere preferably to abiotic surface and *sdiA* deletion did not alter this characteristic, although the amount of adhered bacteria increased (Figure 5A).

We verified that HFC02 strains displayed a long filament-like morphology only in HeLa cells assay (Figure 5B). The *sdiA* complementation was achieved through transformation with pBAD/Myc-His A plasmid, which is regulated by an arabinose induced promoter.

### 3.6. sdiA Diminishes csgD and csgA Transcription

**Figure 6:**
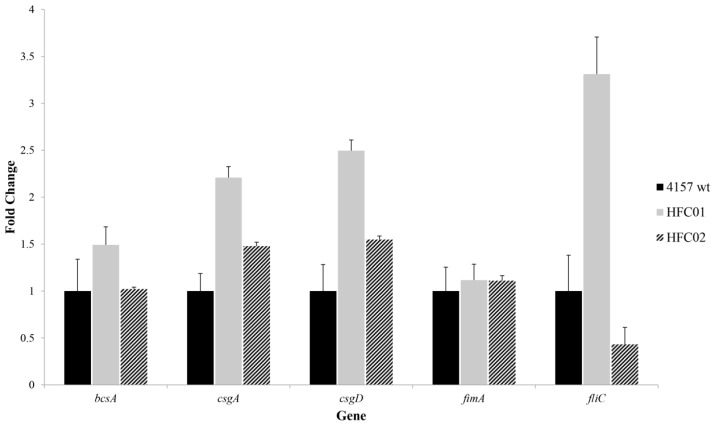
Transcription analysis of biofilm formation related genes (*bcsA*, *csgD*, *csgA*, *fimA* and *fliC*) by quantitative real-time PCR (qRT-PCR), in ONT:H25 (4157 wild type), HFC01 and HFC02 strains. *C_t_* values were normalized by *rpoA* transcription. Error bars represent the standard deviation from triplicates.

qRT-PCR analysis of biofilm formation related genes *csgD*, *csgA*, *bcsA* and *fimA* was performed in wild type, *sdiA* mutant and complemented strains. Transcription levels of *csgD* and *csgA* was significantly greater in ONT:H25 *sdiA* mutant strain (Figure 6). Based on our previous results, we also analyzed the transcription of these biofilm related genes of ONT:H25 wild type and mutant strains in the presence of 3O-C6-DL-HSL. The wild type strain showed a twofold decrease in *csgD* and *fimA* transcription in the presence of this molecule and the mutant strain did not show a statistical significant difference [45].

### 3.7. sdiA Negatively Regulates Motility

**Figure 7:**
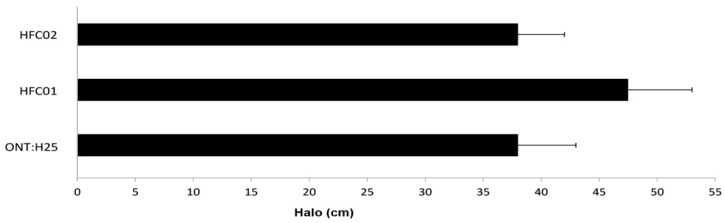
Motility from ONT:H25, HFC01 and HFC02 strains, after 8 h of incubation at 37 °C.

The motility assay depicted an increase of flagellar activity on mutant strains, which can be observed by an increment of the halo diameter (Figure 7). To verify SdiA influence on flagellar motility, *fliC* transcription was evaluated through qRT-PCR. HFC01 displayed more than twofold increase in comparison to wild type strain (Figure 6).

## 4. Discussion

Atypical EPEC has been related to diarrhea outbreaks and sporadic cases of persistent diarrhea worldwide [46,47,48,49]. One of the reasons for the spread of outbreaks and the association with persistent diarrhea may be the ability to form biofilm either in the environment or within the animal host. It has been shown that aEPEC is capable of forming biofilm at temperatures of 26 °C and 37 °C [10,11]. Culler et al. [11], also demonstrated that biofilm formation by aEPEC is not related to serotype, adherence pattern on epithelial cells or antibiotic resistance profiles, highlighting the great heterogeneity of this pathotype [4,5].

The ability to form biofilm is an important factor for the survival of the microorganism. Microbial biofilms are populations of microorganisms adhered to biological and non-biological surfaces, typically surrounded by an extracellular polymeric substance (EPS) matrix. The surrounding matrix acts not only as a protective barrier but also as an adsorbent molecular sieve for nutrients and signaling molecules. Furthermore, it stimulates the development of bacterial cells with distinguished phenotypes given the heterogeneity of the different microenvironments within a biofilm.

Inter or intraspecific cellular interactions inside a biofilm are very complex and involve Quorum sensing mechanisms, which allow bacteria to coordinate its behavior and act as a community to survive and colonize different environments [50].

In several bacterial species, SdiA receptor is involved in the regulation of different genes through Quorum sensing signaling, some of them related to biofilm formation and bacterial adherence. However, in aEPEC there are no reports in the literature concerning the role of this receptor on the regulation of genes related to these structures. Therefore, this study aimed to analyze the influence of SdiA on biofilm formation, as well as its role in the regulation of some biofilm formation related genes (*csgA*, *csgD*, *bcsA*, *fimA* and *fliC*) in one aEPEC strain.

We noticed SdiA influence in the biofilm formation and adhesion to epithelial cells, since *sdiA* mutants showed increased adherence and biofilm formation, suggesting a negative regulation by SdiA, mostly by inhibition of curli fimbriae expression.

Previous studies have reported the increase of adhesion and biofilm formation mediated by curli fimbriae overexpression in O157:H7 EHEC (Sharma et al. [35]). Moreover, the overexpression of *sdiA*, as showed by Lee et al. [27], represses the transcription of curli genes.

Weiss-Muszkatet et al. [10] demonstrated that a *csgFG* mutant O55:H7 aEPEC strain—which participates in curli secretion and assembly—was incapable of forming a pellicle and ring-like structure on the tube wall, evidencing the role of curli in the formation of these structures. In our study, only the HFC01strain formed pellicle on air-liquid interface and this is the only strain positive for curli fimbriae expression, suggesting that this structure might be involved in these phenotypes. These results are associated with *rdar* morphotype, which has been associated with biofilm formation, bacterial aggregation and pellicle formation on air-liquid interface, due to the concomitant expression of curli and cellulose [14].

Another influencing factor on the formation of ring-like structure is the growth temperature. In this study, the mutant strain showed a thicker structure at both 26 °C and 37 °C when compared to wild type and complemented strains. However, this difference was more noticeable at 37 °C.

These results are in agreement to the ones obtained in the assays described above, being possible to associate the increase of fimbriae curli expression at 37 °C with the increase of biofilm by the HFC01 strain.

HFC01 strain showed greater red coloration in comparison with wild type and complemented strains, indicating increased curli fimbriae expression by HFC01 strains. SdiA seems to be important on biofilm regulation acting in different ways depending on the temperature sensing the external conditions and recognizing if the bacteria is in the host at 37 °C, or in the environment at 26 °C and signaling if its best to form biofilms.

SdiA is capable of binding in the DNA sequences of *csgD*, *csgB* and *fimA* [12] and there are at least 15 known transcription factors regulating *sdiA*, including five two-component systems that act as repressors [34]. Suzuki et al. [51] and Houdt et al. [29] demonstrated that the exposure of SdiA to AHL molecules can alter bacterial gene expression pattern depending on the temperature. A plausible explanation for the distinct results verified at different temperatures could be related to an opposite effect of these regulators at 26 °C and 37 °C.

These observations indicated that SdiA might have an important role regulating a variety of *E. coli* phenotypes under different environmental conditions, such as low nutrient availability, temperature changes, pH, among other stress related factors [29]. In fact, those five two-component systems mentioned by Shimada et al. [34] are sensitive to changes in osmolarity (EnvZ-OmpR and RcsABCDF), temperature, cell membrane disorders (RcsABCDF), anaerobic respiration control (ArcBA), high osmolarity, heavy metal exposure (CpxAR), alkaline pH (CpxAR and TorSR), acid pH metabolic control in anaerobic conditions (TorSR). Therefore, it is likely that SdiA probably represses curli fimbriae at 37 °C. On the other hand, when bacteria are exposed to stress conditions, *sdiA* is inhibited alleviating the repressor effect on the expression of these structures, which leads to a rise in the ring-like structure and biofilm formation.

Ring-like structure formation also seems to be a multifactorial process. Hernandes et al. [12] verify that a *fimA* mutant aEPEC strain have lost its ability to form ring-like structures on test tube wall, indicating a role of type 1 fimbriae.

Transcription levels of *fimA*, *csgD* and *csgA* genes were analyzed through qRT-PCR, in order to elucidate the results obtained in the phenotypic assays. HFC01 showed higher levels of transcription of *csgD* and *csgA* genes when compared to the wild type strain, indicating that these components are subjected to Quorum sensing regulation through SdiA, reinforcing the importance of this receptor in aEPEC strains, as a player of QS signaling regulation of these structures and biofilm formation. Therefore, the thick ring-like structure and pellicle formation observed on strain HFC01 is probably a consequence of increased curli fimbriae transcription.

The *fimA* gene not showed variation of transcription when compared to the wild type strain.

Our results, together with those reported by other authors [26,31,32,33,34,35,36] suggest that SdiA, unlike other LuxR homologues, is capable of altering various phenotypes even in the absence of AHL molecules. Although SdiA is functional in the absence of AHL, these molecules enhance its stability and even in the absence of AHL molecules SdiA binds to the *ftsQP2* promoter, involved in cell division [30]. Thus, it appears that AHL intensify the efficiency of SdiA by making it more stable.

We sought to analyze the influence of AHL on biofilm formation of ONT:H25 wild type and *sdiA* mutant strains, which formed a more robust biofilm. We selected 3O-C6-DL-HSL and C8-DL-HSL, since SdiA binds to those AHL molecules [52]. Wild type strains showed a reduced biofilm formation in relation to control strains (no AHL added), especially with C8-DL-HSL at 26 °C and 37 °C after 24 h of incubation. These results corroborate with those reported by Lee et al. [27,28] and Shankar et al. [26] that also verified a reduction of biofilm formation as a consequence of AHL addition. The influence of AHL on SdiA was confirmed by analyzing the mutant strains, which showed no significant difference on biofilm formation. Hughes et al. [32], described possible downregulation of *ler* transcription by AHLs addition in an EHEC strain and lately Nguyen et al. [53], also verified downregulation in *ler*, *espA* and *tir* transcription in an EHEC acyl homoserine synthase (*yenl*^+^) strain, which produces endogenous AHLs.

Therefore, we analyzed the transcription levels of *fimA*, *csgD* and *csgA* in the presence of 3O-C6-DL-HSL. The wild type strains showed approximately a twofold decrease in the transcription of *fimA* and *csgD* when grown in the presence of AHL. The transcription level of *csgA* was slightly lower in comparison to the wild type with no AHL [45]. Similar observations were reported by Shimada et al. [34], which verified that in the presence of AHL the transcription level of *fimA* was decreased. Using CSLM we verified SdiA influence in biofilm structures and architecture.

As stated before, *sdiA* mutant strains formed a thicker biofilm, probably due to the increase of biofilm related structures expression. As expected, *sdiA* mutant strain showed a characteristic mature biofilm, where it was possible to observe pillars, regardless the treatment applied (DMSO, C8-DL-HSL and 3O-C6-DL-HSL). Interestingly, the addition of AHL molecules caused a decrease in biofilm formation of wild type and complemented strains. In these strains, it was not possible to observe the characteristic pillars but only dispersal bacterial cells, corroborating once more SdiA importance regulating biofilm formation.

An interesting point in our results was the observation that, in the presence of epithelial cells, *sdiA* complemented strains showed a remarkable deficiency in cell division. Kanamaru et al. [54] reported that SdiA overexpression positively regulates *ftsQAZ* operon in EHEC and the overexpression of *ftsZ* leads to a tenfold increase in bacterial cell size when in contact with Caco-2 cells. Therefore, the long filament-like cell phenotype observed in our complemented strain could be a result of the overexpression of *sdiA*, since it was cloned in an arabinose induced plasmid. Taken together, these results and the role of SdiA in the regulation of several phenotypes in the absence of AHL molecules, we suggest that SdiA may have the ability to bind to different molecules and trigger distinct cellular responses.

We also analyzed the influence of SdiA on the motility capacity of aEPEC. When compared to wild type, *sdiA* mutant strains presented an increase in motility on semi-solid agar. Similar results were reported by Sharma et al. [35].

Therefore, it seems that *sdiA* deletion drives to biofilm formation in aEPEC strains and this could be benefit to these strains, since the surrounding matrix protects the bacteria from environmental adversities and therefore the presence of SdiA would appear disadvantageous. However, as mentioned early, SdiA is involved on the regulation of several phenotypes depending on environmental conditions.

Besides that, inside a biofilm the bacterial metabolic rate is much lower than in planktonic bacteria, which diminishes the growth rate. In a highly competitive environment, planktonic bacteria would beneficiate from the available nutrients increasing their population density and survival rate. Therefore, the role of SdiA in the regulation of biofilm and biofilm related structures is very important in order to perfectly synchronize its expression when the bacteria experiences stress.

To conclude, all the results observed with *sdiA* mutant strains indicate that SdiA receptor has an important role in the biofilm formation and biofilm related structures in aEPEC. More importantly, SdiA is active and functional either in the presence or absence of AHL molecules. Thus, it is plausible to explain the maintenance of SdiA in *E. coli* genome, even in the absence of an AHL synthase, since this receptor is capable of coordinate several structures regulation without AHL participation. AHL molecules only increase the role of SdiA in the regulation of different promoter regions, which could explain the presumable evolutionary loss of the AHL synthase in *E. coli*.

Besides that, a recent study by Nguyen et al. [36] suggests that SdiA recognizes another molecule present in both prokaryotes and eukaryotes—named 1-octanoyl-rac-glycerol (OCL)—to initiate signaling cascade regulating several genes.

According to our results, SdiA is important to biofilm formation and can sense different environmental conditions, such as osmolarity and temperature, indicating what’s the best *next step* (phenotype) to the bacteria to survive either in the host or in the environment.

## Figures and Tables

**Table 1:** *Escherichia coli* strains and plasmids.

Strains and Plasmids	Description	Reference
Strains		
aEPEC	aEPEC ONT:H25	[37]
HFC01	ONT:H25Δ*sdiA::kan*	Current study
HFC02	HFC01 + pHFC01	Current study
BW 25113	*E. coli* K12 strain harboring plasmid pKD46	[40]
BW 25141	*E. coli* K12 strain harboring plasmid pKD3	[40]
BW 25141	*E. coli* K12 strain harboring plasmid pKD4	[40]
Plasmids		
pHFC01	*sdiA* in pBAD/Myc-His A	Current study
pKD3	λ Red template plasmid (Chloramphenicol)	[40]
pKD4	λ Red template plasmid (Kanamycin)	[40]
pKD46	λ Red helper plasmid	[40]

aEPEC: atypical enteropathogenic *Escherichia coli*.

**Table 2:** Primers used in this study.

Primers	Primer Sequence (5’-3’)	Ref.
*sdiA*-DT	F-CAGTAGCGGCCGCGTAACA	Current study
	R-GAGAATGCGATGGCTTGCAAAAG	
*sdiA*-DL	F-AGCAACCTGCGTCTTATTCGGTGCATTGATTTTTTTCTGCGTGTAGGCTGGAGCTGCTTC	Current study
	R-TATCATTATAAATGATACTCACTCTCAGGGGCGTTGCGGTGAACTAAGGAGGATATTCATATG	
pBAD	F-ATGCCATAGCATTTTTATCC	Current study
	R-GATTTAATCTGTATCAGG	
*sdiA*-*Xho*I	F-GTCGCTCGAGAATGCAGGATACGGATTTTTTC	Current study
*sdiA*-*EcoR*I	R-GTCGGAATTCTCAAATTAAGCCAGTAGCGG	
*bcsA*-RT	F-AGCTCGGCTTCCGTGGC	Current study
	R-TCATTGTTGAGCCAAAGCCT	
*csgA*-RT	F-GATGTTGGTCAGGGCTCAG	[35]
	R-CCACCGAATTGTTTAACTGTC	
*csgD*-RT	F-GGTAACTATCGTTATAACAGCA	Current study
	R-TGCCCAGGAAACCGCTTG	
*fimA*-RT	F-TGTCCCTCAGTTCTACAGCG	[35]
	R-TCCTAACTGAACGGTTTGATC	
*fliC*-RT	F-TCCATCGACAAATTCCGTTCT	[41]
	R-TGGTGACTGCGGAATCCA	
*rpoA*-RT	F-GCGCTCATCTTCTTCCGAAT	[42]
	R-CGCGGTCGTGGTTATGTG	

DT: detection; DL: deletion; Cellulose: *bcsA;* Curli fimbriae: *cgsA* and cgsD; Type I fimbriae: f*imA*; Flagellin: *fliC;* RNA polymerase A: *rpoA*; RT: room temperature.
